# Supplementary figures and images for: Fungal Communities Are Important Determinants of Bacterial Community Composition in Deadwood
Source: mSystems. 2021 Jan 5;6(1):e01017-20. doi: 10.1128/mSystems.01017-20 (PMC7786133; doi:10.1128/mSystems.01017-20)

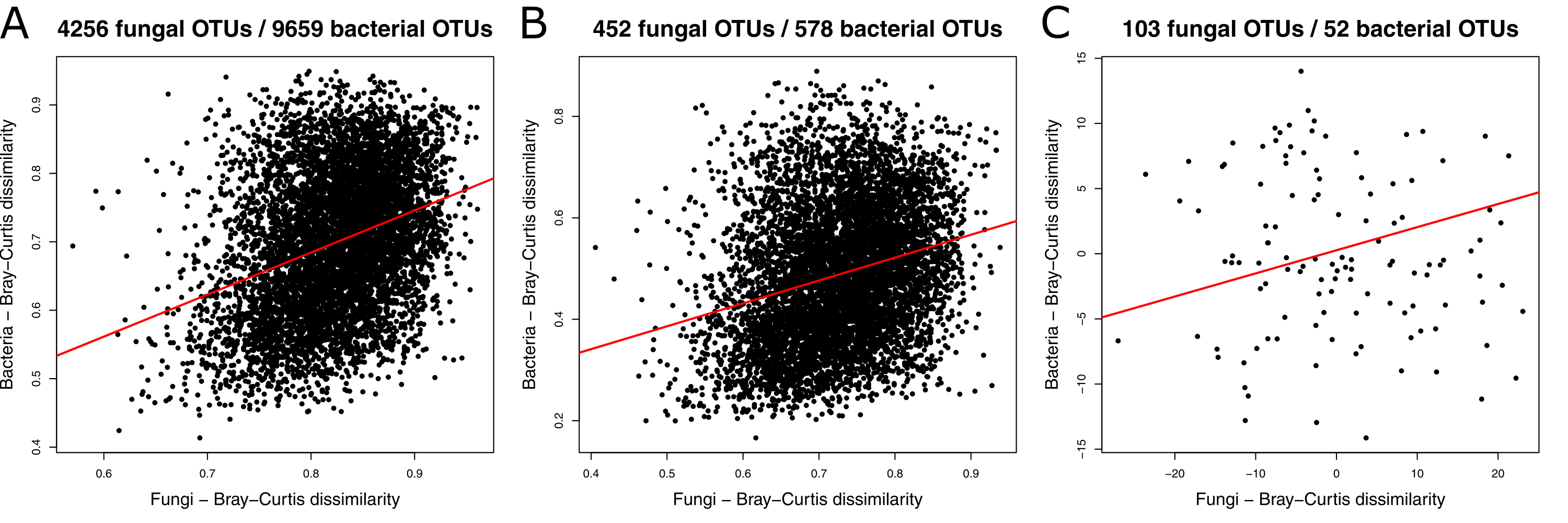

Supplement: FIG S2 [file mSystems.01017-20-sf002.tif]

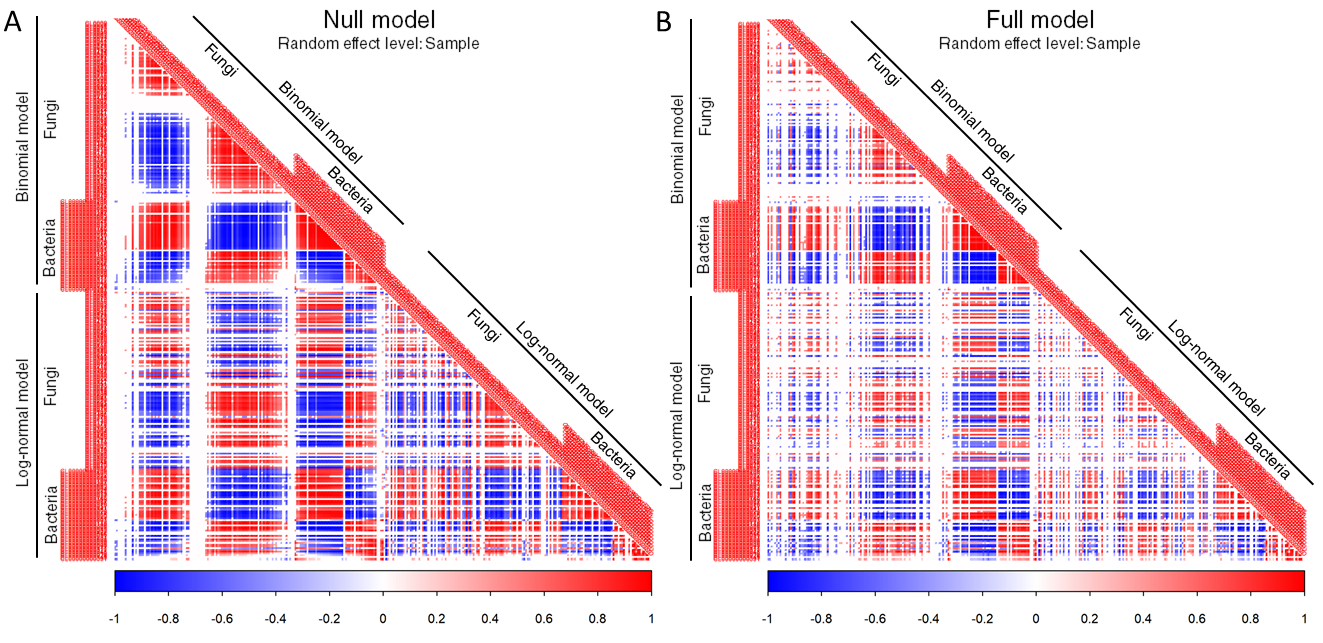

Supplement: FIG S3 [file mSystems.01017-20-sf003.tif]

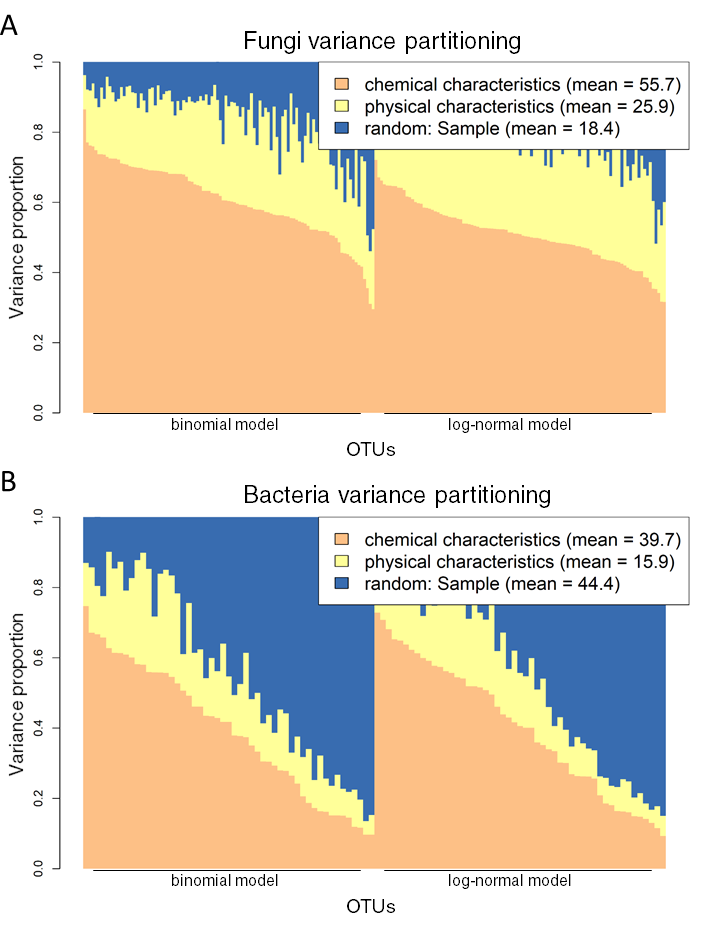

Supplement: FIG S4 [file mSystems.01017-20-sf004.tif]

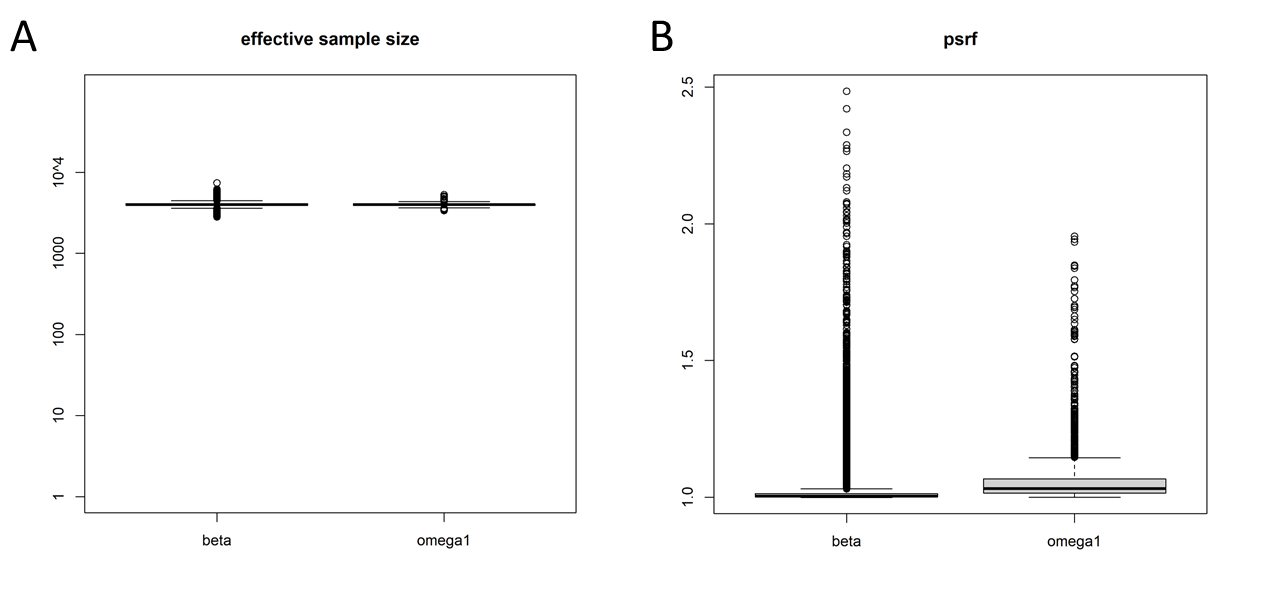

Supplement: FIG S5 [file mSystems.01017-20-sf005.tif]
