# Supplementary material for: Fungal Communities Are Important Determinants of Bacterial Community Composition in Deadwood
Source: mSystems. 2021 Jan 5;6(1):e01017-20. doi: 10.1128/mSystems.01017-20 (PMC7786133; doi:10.1128/mSystems.01017-20)
Supplement: TABLE S1 [file mSystems.01017-20-st001.docx]

|  | Model | Nº of species | Explanatory power | Predictive power | Conditional predictive power |
| --- | --- | --- | --- | --- | --- |
| Fungi occurrence | 1 | 103 | 0.652 | 0.570 | 0.593 |
| Fungi occurrence | 2 | 103 | 0.749 | 0.600 | 0.602 |
| Bacteria occurrence | 1 | 51 | 0.835 | 0.573 | 0.664 |
| Bacteria occurrence | 2 | 51 | 0.869 | 0.650 | 0.669 |
| Fungi abundance | 1 | 103 | 0.141 | -0.028 | 0.005 |
| Fungi abundance | 2 | 103 | 0.312 | 0.034 | 0.040 |
| Bacteria abundance | 1 | 51 | 0.340 | 0.004 | 0.064 |
| Bacteria abundance | 2 | 51 | 0.450 | 0.071 | 0.087 |
