# Supplementary material for: Fungal Communities Are Important Determinants of Bacterial Community Composition in Deadwood
Source: mSystems. 2021 Jan 5;6(1):e01017-20. doi: 10.1128/mSystems.01017-20 (PMC7786133; doi:10.1128/mSystems.01017-20)
Supplement: TABLE S2 [file mSystems.01017-20-st002.docx]

| **Tree sp.** | **Decay class (yr)** | **DBH (cm)** | **Latitude** | **Longitude** | **pH** | **N (%)** | **C (%)** | **Water content (%)** | **Lignin (%)** | **CN ratio** |
| --- | --- | --- | --- | --- | --- | --- | --- | --- | --- | --- |
| *Abies alba* | 5-15 | 78 | 48.66728 | 14.71042 | 4.45 | 0.48 | 44.76 | 80.53941 | 52.8 | 93.25 |
| *Fagus sylvatica* | 5-15 | 83 | 48.6673 | 14.70959 | 4.95 | 0.226 | 47.09 | 57.88949 | 24.25 | 208.3628 |
| *Fagus sylvatica* | 16-38 | 30 | 48.66687 | 14.70943 | 3.85 | 0.275 | 47.15 | 57.09942 | 32.65 | 171.4545 |
| *Picea abies* | <5 | 34 | 48.66697 | 14.70998 | 4.17 | 0.12 | 49.43 | 54.7619 | 29 | 411.9167 |
| *Picea abies* | <5 | 60 | 48.66695 | 14.71021 | 4 | 0.134 | 53.33 | 59.3719 | 38.4 | 397.9851 |
| *Fagus sylvatica* | <5 | 60 | 48.66638 | 14.70988 | 4.97 | 0.505 | 47.09 | 84.14593 | 40.4 | 93.24752 |
| *Fagus sylvatica* | <5 | 42 | 48.66631 | 14.70949 | 5.42 | 0.21 | 46.25 | 28.17781 | 26.25 | 220.2381 |
| *Fagus sylvatica* | <5 | 58 | 48.66641 | 14.70943 | 5.95 | 0.136 | 45.05 | 40.0201 | 25.5 | 331.25 |
| *Abies alba* | 16-38 | 90 | 48.66642 | 14.70906 | 3.45 | 0.17 | 50.09 | 74.41336 | 39 | 294.6471 |
| *Picea abies* | 5-15 | 81 | 48.66657 | 14.70876 | 4.37 | 0.074 | 50.57 | 52.05079 | 31.75 | 683.3784 |
| *Picea abies* | 5-15 | 60 | 48.66702 | 14.70809 | 4.17 | 0.085 | 51.11 | 47.74612 | 33.6 | 601.2941 |
| *Picea abies* | 16-38 | 83 | 48.66719 | 14.70816 | 4.55 | 0.234 | 43.49 | 72.96779 | 34.6 | 185.8547 |
| *Picea abies* | 5-15 | 53 | 48.66749 | 14.70847 | 4.29 | 0.086 | 51.95 | 63.09621 | 32.6 | 604.0698 |
| *Picea abies* | 16-38 | 60 | 48.66664 | 14.70733 | 3.84 | 0.264 | 53.03 | 75.069 | 43 | 200.8712 |
| *Picea abies* | 5-15 | 99 | 48.66676 | 14.70662 | 3.63 | 0.258 | 51.77 | 49.58564 | 37 | 200.6589 |
| *Picea abies* | >38 | 70 | 48.66713 | 14.70661 | 3.49 | 0.584 | 50.39 | 64.88746 | 46.05 | 86.28425 |
| *Picea abies* | 5-15 | 65 | 48.6672 | 14.70614 | 3.86 | 0.742 | 54.29 | 68.50347 | 62.65 | 73.16712 |
| *Picea abies* | <5 | 74 | 48.66725 | 14.70584 | 4.06 | 0.092 | 51.23 | 28.06574 | 29.6 | 556.8478 |
| *Picea abies* | 16-38 | 80 | 48.66746 | 14.70616 | 3.54 | 0.43 | 53.75 | 76.04062 | 61.1 | 125 |
| *Picea abies* | <5 | 48 | 48.66694 | 14.7056 | 4.88 | 0.092 | 49.01 | 36.35093 | 33.2 | 532.7174 |
| *Abies alba* | 16-38 | 100 | 48.66664 | 14.70587 | 3.48 | 0.562 | 51.53 | 82.38201 | 30.35 | 91.69039 |
| *Picea abies* | <5 | 35 | 48.66684 | 14.70519 | 4.09 | 0.111 | 51.65 | 33.99868 | 30.9 | 465.3153 |
| *Picea abies* | 16-38 | 40 | 48.66718 | 14.70489 | 4.24 | 0.263 | 49.19 | 76.57719 | 34.3 | 187.0342 |
| *Picea abies* | 5-15 | 30 | 48.66759 | 14.70529 | 4.89 | 0.059 | 48.71 | 21.9854 | 28.15 | 825.5932 |
| *Abies alba* | 16-38 | 40 | 48.66775 | 14.70485 | 3.62 | 0.391 | 51.35 | 59.3719 | 38.4 | 131.3299 |
| *Fagus sylvatica* | <5 | 58 | 48.66807 | 14.70471 | 4.5 | 0.153 | 47.75 | 55.85996 | 29.65 | 312.0915 |
| *Abies alba* | >38 | 43 | 48.66758 | 14.70418 | 3.45 | 0.699 | 46.19 | 83.21789 | 52 | 66.08011 |
| *Fagus sylvatica* | 16-38 | 70 | 48.66763 | 14.70367 | 4.69 | 1.389 | 44.94 | 77.37022 | 57.25 | 32.35421 |
| *Fagus sylvatica* | 16-38 | 60 | 48.66751 | 14.7037 | 4.62 | 0.615 | 50.03 | 84.26794 | 47.95 | 81.34959 |
| *Picea abies* | 16-38 | 30 | 48.66749 | 14.70372 | 4.04 | 0.905 | 48.71 | 72.69138 | 46.1 | 53.8232 |
| *Fagus sylvatica* | 5-15 | 95 | 48.66689 | 14.70354 | 5 | 0.199 | 48.11 | 32.79549 | 20.2 | 241.7588 |
| *Fagus sylvatica* | 16-38 | 50 | 48.66697 | 14.70376 | 4.01 | 0.376 | 52.37 | 73.72851 | 38.65 | 139.2819 |
| *Fagus sylvatica* | 16-38 | 70 | 48.66673 | 14.70356 | 3.53 | 0.989 | 50.93 | 70.21298 | 48.15 | 51.49646 |
| *Abies alba* | 16-38 | 70 | 48.66657 | 14.7041 | 3.46 | 0.759 | 55.19 | 75.13561 | 50.85 | 72.7141 |
| *Abies alba* | <5 | 100 | 48.66632 | 14.70361 | 3.85 | 0.139 | 55.13 | 78.02245 | 37.65 | 396.6187 |
| *Abies alba* | >38 | 60 | 48.66594 | 14.70323 | 3.73 | 0.149 | 53.99 | 68.82881 | 35.1 | 362.349 |
| *Picea abies* | >38 | 70 | 48.66546 | 14.70517 | 4.65 | 0.39 | 52.25 | 53.8874 | 30.35 | 133.9744 |
| *Fagus sylvatica* | 16-38 | 50 | 48.66583 | 14.70536 | 4.15 | 0.91 | 48.65 | 86.43184 | 29.2 | 53.46154 |
| *Picea abies* | >38 | 70 | 48.66616 | 14.7056 | 3.72 | 0.157 | 54.05 | 62.17515 | 44.2 | 344.2675 |
| *Abies alba* | 16-38 | 96 | 48.66595 | 14.70583 | 3.45 | 0.12 | 52.79 | 67.70637 | 33.55 | 439.9167 |
| *Picea abies* | >38 | 80 | 48.6664 | 14.70616 | 3.8 | 0.19 | 53.51 | 79.9834 | 47.35 | 281.6316 |
| *Picea abies* | >38 | 60 | 48.66616 | 14.70623 | 3.69 | 0.544 | 50.03 | 80.08282 | 32.1 | 91.96691 |
| *Fagus sylvatica* | <5 | 55 | 48.66597 | 14.70663 | 4.83 | 0.275 | 48.71 | 65.54653 | 30.4 | 177.1273 |
| *Abies alba* | 5-15 | 54 | 48.66604 | 14.70686 | 4.66 | 0.122 | 44.09 | 32.48676 | 35.3 | 361.3934 |
| *Picea abies* | 16-38 | 60 | 48.6658 | 14.70653 | 4.58 | 0.662 | 39.41 | 77.3929 | 52.7 | 59.53172 |
| *Fagus sylvatica* | 5-15 | 66 | 48.66373 | 14.70715 | 5.37 | 0.141 | 40.01 | 48.17262 | 28.2 | 283.7589 |
| *Abies alba* | 5-15 | 66 | 48.66559 | 14.70696 | 4.25 | 0.106 | 43.01 | 49.04262 | 34.45 | 405.7547 |
| *Fagus sylvatica* | 5-15 | 34 | 48.66552 | 14.70688 | 5.43 | 0.131 | 42.05 | 36.80691 | 26.55 | 320.9924 |
| *Picea abies* | 5-15 | 47 | 48.66523 | 14.70667 | 3.36 | 0.075 | 42.35 | 29.15737 | 34.05 | 564.6667 |
| *Picea abies* | 5-15 | 37 | 48.66515 | 14.70649 | 3.16 | 0.062 | 41.33 | 32.72009 | 35.95 | 666.6129 |
| *Fagus sylvatica* | 5-15 | 59 | 48.66584 | 14.70785 | 4.77 | 0.347 | 43.13 | 59.3719 | 34 | 124.2939 |
| *Abies alba* | >38 | 80 | 48.66622 | 14.70755 | 3.46 | 0.267 | 50.75 | 79.86602 | 51.4 | 190.0749 |
| *Fagus sylvatica* | 5-15 | 61 | 48.66555 | 14.70869 | 4.9 | 0.154 | 38.69 | 42.8305 | 14.6 | 251.2338 |
| *Fagus sylvatica* | 5-15 | 70 | 48.66598 | 14.70922 | 4.41 | 0.589 | 44.99 | 86.78725 | 38.75 | 76.3837 |
| *Abies alba* | 5-15 | 60 | 48.6657 | 14.70937 | 4.38 | 0.075 | 44.21 | 30.73993 | 23.6 | 589.4667 |
| *Fagus sylvatica* | 16-38 | 100 | 48.66487 | 14.70905 | 4.12 | 0.195 | 44.27 | 69.45366 | 28.3 | 227.0256 |
| *Fagus sylvatica* | >38 | 70 | 48.6641 | 14.70953 | 4.56 | 1.174 | 47.09 | 86.66903 | 59.9 | 40.11073 |
| *Fagus sylvatica* | 16-38 | 90 | 48.66431 | 14.70805 | 4.38 | 0.483 | 41.21 | 74.87196 | 33.2 | 85.32091 |
| *Abies alba* | 5-15 | 95 | 48.66404 | 14.70659 | 3.84 | 0.099 | 44.33 | 62.60404 | 37.95 | 447.7778 |
| *Abies alba* | 5-15 | 45 | 48.66401 | 14.70576 | 4.54 | 0.108 | 40.25 | 37.01896 | 32.8 | 372.6852 |
| *Picea abies* | 5-15 | 43 | 48.66467 | 14.70595 | 3.8 | 0.061 | 47.09 | 31.63632 | 30 | 771.9672 |
| *Abies alba* | 5-15 | 83 | 48.66526 | 14.70581 | 4.3 | 0.145 | 45.11 | 46.29056 | 28.95 | 311.1034 |
| *Abies alba* | 5-15 | 70 | 48.66533 | 14.70516 | 3.73 | 0.161 | 45.35 | 61.35722 | 30.6 | 281.677 |
| *Picea abies* | >38 | 40 | 48.66546 | 14.70433 | 4.05 | 0.132 | 42.65 | 48.96614 | 34.3 | 323.1061 |
| *Picea abies* | 5-15 | 71 | 48.66542 | 14.70385 | 4.6 | 0.073 | 43.79 | 46.49058 | 31.25 | 599.863 |
| *Picea abies* | 16-38 | 100 | 48.6648 | 14.70333 | 4.11 | 0.339 | 49.31 | 46.23229 | 69.35 | 145.4572 |
| *Abies alba* | >38 | 65 | 48.6645 | 14.70445 | 3.13 | 0.101 | 45.47 | 47.05512 | 31.9 | 450.198 |
| *Fagus sylvatica* | 5-15 | 75 | 48.66431 | 14.70447 | 5.52 | 0.178 | 38.45 | 52.16687 | 24.25 | 216.0112 |
| *Abies alba* | 5-15 | 80 | 48.66414 | 14.7043 | 4.66 | 0.088 | 42.77 | 54.63116 | 40.4 | 486.0227 |
| *Abies alba* | 16-38 | 60 | 48.66435 | 14.70461 | 4.33 | 0.209 | 45.41 | 52.10457 | 34.95 | 217.2727 |
| *Fagus sylvatica* | <5 | 41 | 48.66295 | 14.70871 | 5.62 | 0.122 | 40.07 | 45.31892 | 18.55 | 328.4426 |
| *Fagus sylvatica* | 5-15 | 45 | 48.66414 | 14.70269 | 4.74 | 0.14 | 40.07 | 45.86764 | 23.6 | 286.2143 |
| *Picea abies* | >38 | 70 | 48.66386 | 14.70278 | 3.93 | 0.374 | 50.75 | 80.10062 | 75.75 | 135.6952 |
| *Picea abies* | <5 | 35 | 48.66369 | 14.70285 | 4.29 | 0.059 | 43.55 | 19.21909 | 29.45 | 738.1356 |
| *Picea abies* | <5 | 45 | 48.6638 | 14.70316 | 3.82 | 0.107 | 45.23 | 30.61186 | 34.1 | 422.7103 |
| *Picea abies* | 16-38 | 40 | 48.66396 | 14.70313 | 3.82 | 0.543 | 47.57 | 78.52398 | 77.05 | 87.60589 |
| *Picea abies* | 5-15 | 90 | 48.66401 | 14.70318 | 3.44 | 0.299 | 49.85 | 82.35949 | 61.35 | 166.7224 |
| *Fagus sylvatica* | 16-38 | 80 | 48.66357 | 14.7043 | 3.54 | 0.452 | 43.97 | 80.20641 | 24.95 | 97.27876 |
| *Picea abies* | 16-38 | 70 | 48.66383 | 14.70478 | 3.88 | 0.152 | 49.07 | 44.82294 | 31.65 | 322.8289 |
| *Abies alba* | 16-38 | 90 | 48.6636 | 14.70538 | 3.46 | 0.65 | 47.39 | 84.4499 | 67.25 | 72.90769 |
| *Abies alba* | 16-38 | 90 | 48.66379 | 14.7065 | 3.15 | 0.189 | 46.37 | 61.77045 | 46 | 245.3439 |
| *Fagus sylvatica* | 5-15 | 39 | 48.66342 | 14.70723 | 4.61 | 0.195 | 41.15 | 40.28628 | 24.3 | 211.0256 |
| *Fagus sylvatica* | 16-38 | 80 | 48.66365 | 14.70741 | 3.32 | 0.152 | 44.99 | 71.31698 | 33.1 | 295.9868 |
| *Abies alba* | 5-15 | 100 | 48.66342 | 14.70796 | 3.99 | 0.098 | 46.85 | 38.03801 | 40.1 | 478.0612 |
| *Fagus sylvatica* | 5-15 | 80 | 48.66318 | 14.70786 | 5.54 | 0.187 | 39.17 | 38.09702 | 19.65 | 209.4652 |
| *Picea abies* | <5 | 52 | 48.66295 | 14.70905 | 4.66 | 0.082 | 40.55 | 40.96577 | 34.25 | 494.5122 |
| *Picea abies* | <5 | 36 | 48.66296 | 14.70889 | 4.49 | 0.067 | 45.77 | 32.27506 | 34.4 | 683.1343 |
| *Picea abies* | <5 | 56 | 48.66564 | 14.70499 | 3.47 | 0.07 | 43.19 | 41.69016 | 29.05 | 617 |
| *Picea abies* | 16-38 | 50 | 48.66568 | 14.70455 | 3.64 | 0.233 | 51.77 | 74.55909 | 38.4 | 222.1888 |
| *Picea abies* | >38 | 90 | 48.66599 | 14.70488 | 3.13 | 0.082 | 45.11 | 47.43756 | 33.6 | 550.122 |
| *Picea abies* | >38 | 60 | 48.66614 | 14.70478 | 3.78 | 0.315 | 49.19 | 71.29705 | 63.3 | 156.1587 |
| *Picea abies* | 16-38 | 70 | 48.66589 | 14.70486 | 3.61 | 0.629 | 45.17 | 69.13931 | 57.6 | 71.8124 |
| *Fagus sylvatica* | 16-38 | 40 | 48.66345 | 14.70494 | 4.58 | 0.388 | 43.19 | 60.21794 | 33.8 | 111.3144 |
| *Abies alba* | 5-15 | 90 | 48.66388 | 14.70719 | 5.46 | 0.137 | 42.95 | 44.59479 | 34.55 | 313.5036 |
| *Fagus sylvatica* | <5 | 55 | 48.66395 | 14.70746 | 4.61 | 0.31 | 41.75 | 74.47766 | 32.8 | 134.6774 |
| *Abies alba* | 16-38 | 80 | 48.66366 | 14.70659 | 3.57 | 1.746 | 42.11 | 70.10206 | 55.7 | 24.11798 |
| *Abies alba* | 16-38 | 70 | 48.66361 | 14.70592 | 3.47 | 0.565 | 47.69 | 82.10267 | 41.5 | 84.40708 |
| *Abies alba* | 5-15 | 40 | 48.6637 | 14.70585 | 4.64 | 0.096 | 45.35 | 58.13951 | 35.25 | 472.3958 |
| *Abies alba* | >38 | 130 | 48.66379 | 14.70667 | 3.99 | 0.621 | 48.83 | 71.48507 | 57.7 | 78.63124 |
| *Fagus sylvatica* | >38 | 70 | 48.66386 | 14.70564 | 3.5 | 0.987 | 45.36 | 85.77559 | 43.45 | 45.95745 |
| *Abies alba* | 5-15 | 88 | 48.66389 | 14.70523 | 6.36 | 0.124 | 40.65 | 44.29119 | 39.4 | 327.8226 |
| *Abies alba* | >38 | 70 | 48.66373 | 14.70503 | 3.58 | 0.871 | 41.38 | 72.55558 | 42.65 | 47.50861 |
| *Picea abies* | >38 | 46 | 48.66569 | 14.70432 | 3.78 | 0.148 | 44.58 | 47.67369 | 22.7 | 301.2162 |
| *Fagus sylvatica* | 5-15 | 30 | 48.6647 | 14.70873 | 4.52 | 0.157 | 41.62 | 43.07983 | 22.75 | 265.0955 |
| *Abies alba* | 16-38 | 70 | 48.66439 | 14.70537 | 3.65 | 0.517 | 48.02 | 85.93392 | 57.1 | 92.88201 |
| *Fagus sylvatica* | 16-38 | 40 | 48.66498 | 14.70766 | 4.03 | 0.63 | 39.74 | 81.59704 | 36.5 | 63.07937 |
| *Fagus sylvatica* | 5-15 | 93 | 48.66527 | 14.70827 | 5.74 | 0.138 | 41.25 | 40.01066 | 20.15 | 298.913 |
| *Fagus sylvatica* | <5 | 65 | 48.66532 | 14.7088 | 5.31 | 0.402 | 41.62 | 85.46673 | 12.5 | 103.5323 |
| *Fagus sylvatica* | >38 | 48 | 48.6673 | 14.70437 | 4.94 | 0.662 | 41.31 | 65.79741 | 45.1 | 62.40181 |
| *Fagus sylvatica* | >38 | 80 | 48.6667 | 14.70776 | 3.99 | 0.95 | 43.19 | 73.28456 | 56.4 | 45.46316 |
| *Fagus sylvatica* | >38 | 80 | 48.66657 | 14.70783 | 3.55 | 0.158 | 42.95 | 26.2844 | 40 | 271.8354 |
| *Fagus sylvatica* | <5 | 85 | 48.66696 | 14.70483 | 4.09 | 0.076 | 44.64 | 44.20712 | 34.25 | 587.3684 |
| *Picea abies* | 5-15 | 76 | 48.6661 | 14.70736 | 4.08 | 0.083 | 44.27 | 45.83515 | 29.8 | 533.3735 |
| *Abies alba* | >38 | 70 | 48.66603 | 14.70749 | 3.88 | 0.222 | 43.55 | 73.10194 | 32.8 | 196.1712 |
| *Abies alba* | <5 | 60 | 48.66356 | 14.7054 | 4.55 | 0.44 | 47.42 | 64.23567 | 52.05 | 107.7727 |
| *Abies alba* | 16-38 | 30 | 48.66389 | 14.70622 | 4.15 | 0.391 | 46.81 | 73.1421 | 51.65 | 119.7187 |
| *Abies alba* | 5-15 | 75 | 48.66636 | 14.7065 | 4.26 | 0.151 | 43.49 | 54.59364 | 37.35 | 288.0132 |
| *Abies alba* | 16-38 | 50 | 48.6638 | 14.70722 | 3.78 | 0.179 | 45.48 | 72.73424 | 28.5 | 254.0782 |
